# Supplementary material for: Predicting biological pathways of chemical compounds with a profile-inspired approach
Source: BMC Bioinformatics. 2021 Jun 12;22:320. doi: 10.1186/s12859-021-04252-y (PMC8199418; doi:10.1186/s12859-021-04252-y)
Supplement: Supplementary file 1 — Additional file 1: Table S1. Composition of databases and fingerprints used. Table S6. Precision (or positive predictive value, PPV) and sensitivity of the prediction of a dataset of 1313 compounds using TrackSM and iFragMent methods. Figure S1. The associated p-value resulting from iFragMent predictions can be used to reduce the FDR. Figure S2. iFragMent server predictions for histidine (C00135 in KEGG). Figure S3. iFragMent server predictions for phospho-DPD (C20959 in KEGG). [file 12859_2021_4252_MOESM1_ESM.docx]

**Supplementary Table 1.** Composition of databases and fingerprints used.

| **Database** | **Length of vectors** | **Number of vectors** | **Number of pathways** | **Compound-Pathway Pairs** |
| --- | --- | --- | --- | --- |
| KEGG | 25191 | 5236 | 214 | 8261 |
| EnviPath | 8255 | 1345 | 65 | 931 |
| Reactome | 10950 | 1326 | 249 | 4536 |
| SMPDB | 11666 | 1408 | 333 | 9031 |

**Supplementary Figure 1**. The associated p-value resulting from iFragMent predictions can be used to reduce the FDR.


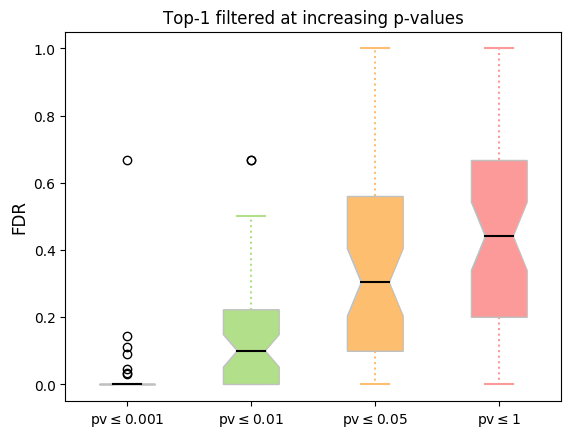


**Supplementary Figure 2.** iFragMent server predictions for histidine (C00135 in KEGG)


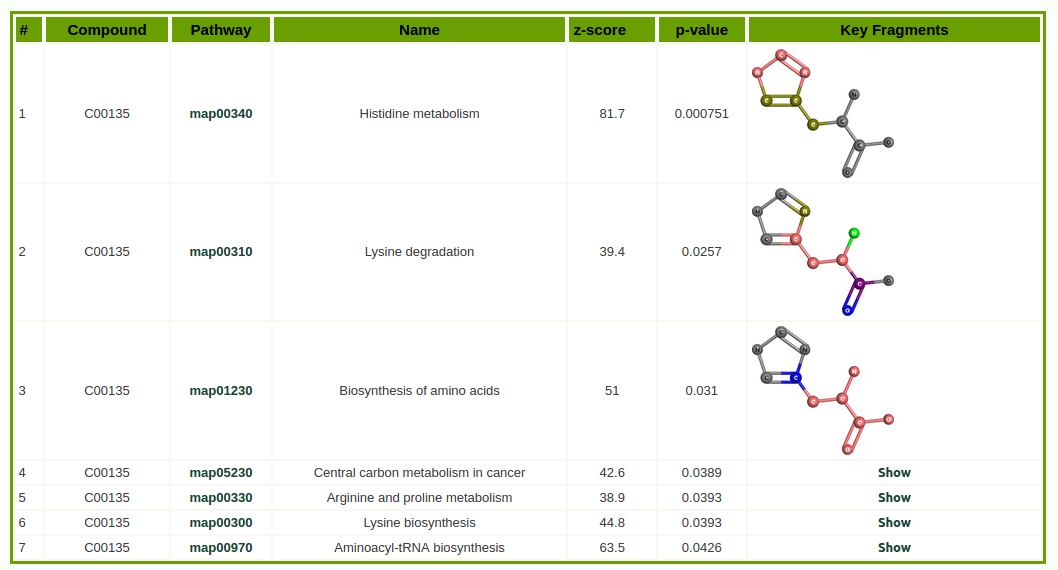


**Supplementary Figure 3.** iFragMent server predictions for phospho-DPD (C20959 in KEGG)


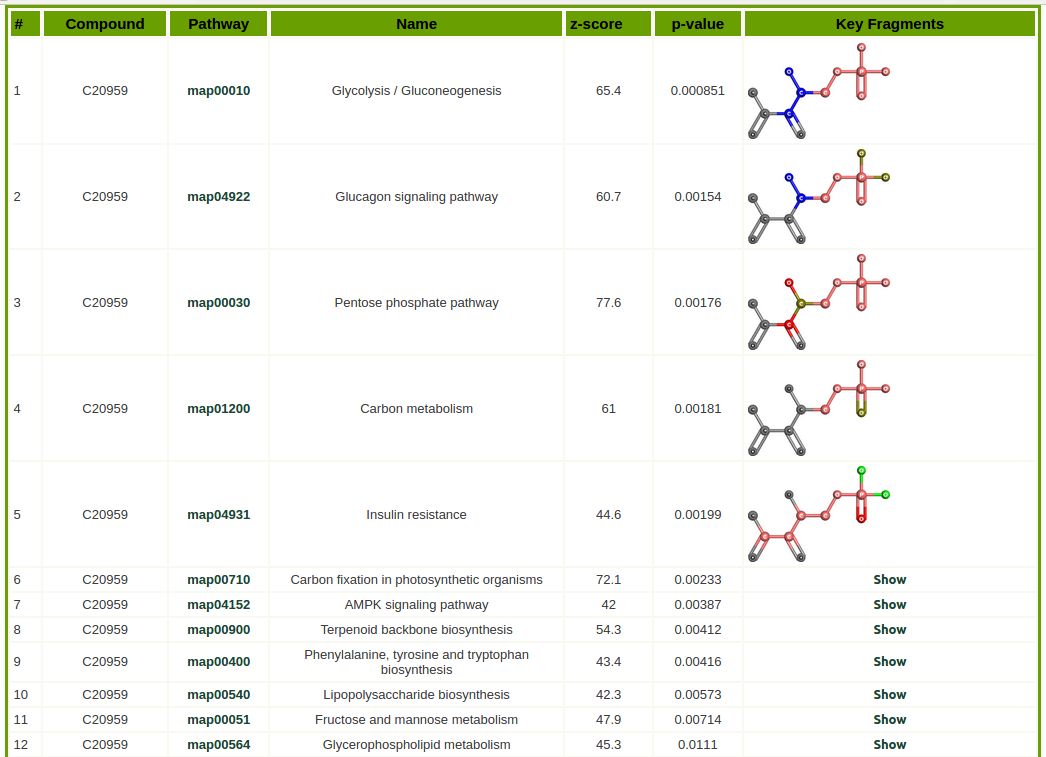


**Supplementary Table 6.** Precision (or positive predictive value, PPV) and sensitivity of the prediction of a dataset of 1313 compounds using TrackSM and iFragMent methods.

| **method** | **ppv** | **sensitivity** | **tp** | **fp** | **fn** | **number of predictions** |
| --- | --- | --- | --- | --- | --- | --- |
| TrackSM | 0.26 | 0.92 | 346 | 967 | 29 | 1313 |
| iFragMent | 0.42 | 0.92 | 548 | 765 | 48 | 1313 |
